# Field Performance of Novel Citrus Rootstocks Grafted with ‘Valencia’ Orange and Their Response to Systemic Delivery of Oxytetracycline

**DOI:** 10.3390/plants14193020

**Published:** 2025-09-29

**Authors:** Caroline Tardivo, Gabriel Pugina, Kim D. Bowman, Ute Albrecht

**Affiliations:** 1 Southwest Florida Research and Education Center, Horticultural Sciences Department, Institute of Food and Agricultural Sciences, University of Florida, Immokalee, FL 34142, USA; gabrieldecastro@ufl.edu; 2 US Horticultural Research Laboratory, Agricultural Research Service, US Department of Agriculture, Fort Pierce, FL 34945, USA; kim.bowman@usda.gov

**Keywords:** Huanglongbing (HLB), *Candidatus* Liberibacter asiaticus, trunk injection, SuperSour, disease management, fibrous roots, integrated management

## Abstract

The global citrus industry faces unprecedented challenges due to Huanglongbing (HLB), which is associated with the bacterial pathogen *Candidatus* Liberibacter asiaticus (*C*Las). This study evaluates the field performance of 11 rootstocks, grafted with ‘Valencia’ orange (*Citrus sinensis*), under Florida’s HLB-endemic production conditions, while also examining the impact of systemic applications of oxytetracycline (OTC) via trunk injection. Mature trees received annual OTC injections and were assessed over two production seasons. In year 1, OTC-treated trees exhibited significant improvements regardless of the rootstock, including a 36% increase in yield, an 11% increase in juice TSS, and reduced leaf bacterial titers. During year 2, the positive effects of OTC were sustained, or even enhanced. *C*Las titers were reduced in both leaves and roots; yield increased by 70%; and fruit weight, juice color, and TSS also improved significantly. Moreover, OTC-injected trees exhibited a larger percentage of finer roots compared to non-injected trees. US-1688 and US-1672, both hybrids of *C. maxima* ‘Hirado’ and *C. reticulata* ‘Cleopatra’, emerged as the most productive rootstocks. These results demonstrate the importance of rootstock selection for sustainable citrus cultivation while highlighting the benefits of integrating the systemic delivery of OTC to manage HLB and maximize the resilience of citrus.

## 1. Introduction

Citrus rootstocks are key to enhancing grafted tree performance and resilience, particularly for managing devastating diseases like Huanglongbing (HLB), also known as citrus greening. Associated with the bacterium *Candidatus* Liberibacter asiaticus (*C*Las) in Florida, HLB severely reduces fruit yield and quality, eventually leading to tree decline [1,2,3,4]. HLB has been documented in Florida since 2005 and has become endemic and increasingly damaging [4,5]. One of the most important strategies to prevent the disease is controlling its vector, the Asian Citrus psyllid; however, this has become problematic due to rising costs and resistance to the available insecticides [6,7]. Since no commercial sweet orange scion exhibits natural resistance to HLB [8,9,10,11], selecting superior rootstocks has become a critical strategy to mitigate the disease’s impact [12,13,14]. Rootstock choice directly influences tree vigor, precocity, fruit quality, yield, and disease tolerance, with variations in root architecture and other factors affecting aboveground traits [15,16].

One of the earliest manifestations of *C*Las infection is fibrous root loss [17,18], which can rapidly compromise the water and nutrient status of the whole tree, especially in young trees. Several rootstock cultivars have shown tolerance to HLB, improving the performance of the grafted scion [12,19,20,21]. In particular, citrandarins, hybrids of mandarin (*C. reticulata*) and trifoliate orange (*Poncirus trifoliata*), have emerged as some of the most tolerant rootstocks [12,19] and are widely used in citrus production in Florida. In response to the need for superior rootstocks, the USDA rootstock breeding program introduced the SuperSour strategy, a multi-faceted approach addressing limitations in traditional breeding [22]. By eliminating the need for apomictic seed reproduction and leveraging underutilized germplasm (e.g., *C. maxima*), combined with molecular marker development [23], this strategy accelerates the development of new rootstocks with better adaptation to abiotic and biotic stresses, particularly HLB.

In parallel with rootstock development, trunk injection has emerged as a targeted strategy for managing HLB [24,25,26,27]. This strategy, which involves delivering therapeutic compounds directly into the tree’s vascular system, bypasses the inefficiencies of conventional chemical spraying or soil drenching. Its origins date back centuries [28], and modern adaptations have demonstrated the efficacy of injecting antibiotics such as oxytetracycline (OTC) for reducing *C*Las bacterial titers, improving tree health, and enhancing fruit yield and quality of citrus trees growing under high HLB pressure [26,29]. Trunk injection of antibiotics was previously investigated in the 1970s and 1980s in South Africa and other countries where HLB (also known as citrus greening or likubin) was prevalent [30,31,32] but was not adopted for large-scale applications. Due to the severity of HLB in Florida, trunk injection of OTC was approved for commercial use in the state in 2022 and has been widely adopted ever since.

The objective of this study was to determine the effectiveness of combining rootstock selection with the systemic delivery of OTC. Specifically, this work aimed to (1) assess the field performance of nine novel rootstocks; (2) evaluate the impact of OTC trunk injections to reduce pathogen pressure; and (3) assess the potential of this integrated strategy to enhance disease tolerance and promote sustainable citrus production. Minimizing chemical inputs, reducing environmental impacts, and improving the resilience and productivity of citrus orchards through this integrated approach may offer a viable option for HLB management under endemic conditions.

## 2. Results

### 2.1. Candidatus Liberibacter asiaticus (CLas) Detection

Four months after the first injection in 2023, OTC significantly increased leaf Ct-values (28.1), indicating reduced *C*Las bacterial load in the leaves of injected trees compared to control trees (22.2) (Table 1). OTC-injected trees also exhibited higher fibrous root Ct-values (33.3) than control (non-injected) trees (31.8), though the difference was not statistically significant. The rootstock cultivar did not influence leaf Ct-values, but significantly influenced fibrous root Ct-values, with US-2132 exhibiting the highest Ct-value (34.6), indicating the lowest bacterial load among rootstocks, and sour orange exhibiting the lowest Ct-value (29.3) and therefore the highest bacterial load. No significant interactions were observed between rootstock cultivar and injection treatment for either leaf or fibrous root Ct-values. Block effects were also not significant.

Four months after the second injection in 2024, OTC significantly increased leaf Ct-values (28.5) compared to control trees (21.6). OTC-injected trees also exhibited significantly higher fibrous root Ct-values (31.9) compared to control trees (29.1) (Table 1). The rootstock cultivar did not influence leaf Ct-values but significantly influenced fibrous root Ct-values. US-2132 exhibited the highest Ct-value (33.3), while sour orange had the lowest (27.2), reflecting the highest bacterial load in the roots. No significant interactions were observed between rootstock cultivar and injection treatment for either leaf or fibrous root Ct-values. Block effects were also not significant.

### 2.2. Tree Growth

In November 2024, 18 months after the first OTC injection, there were significant injection treatment and rootstock cultivar effects on tree growth (Table S1). Tree height was not significantly influenced by injection treatment but by rootstock cultivar. Sour orange and US-1688 induced the tallest trees (2.3 m and 2.2 m, respectively), while US-2132 and US-2137 induced the shortest (1.9 m). OTC-injected trees had significantly larger canopy volumes (3.1 m^3^) than control trees (2.7 m^3^). The rootstock cultivar also significantly influenced canopy volume, with sour orange and US-1688 inducing the largest canopy volumes (3.8 m^3^ and 3.9 m^3^, respectively) and US-2111, US-2132, and US-2137 the smallest (2.2–2.4 m^3^). OTC-injected trees also exhibited larger scion trunk circumferences (30.0 cm) than control trees (28.7 cm), though the difference was marginally significant (*p* = 0.0585). Sour orange induced the largest scion trunk circumference (34.0 cm), followed by US-1688 (33.4 cm), while Swingle, US-1673, US-1676, US-2132, and US-2137 induced the smallest (26.2–28.5 cm). OTC injections did not significantly affect the rootstock circumference. In contrast, rootstock trunk circumferences varied significantly among rootstocks, with Swingle exhibiting the largest circumference (44.6 cm) and US-1673, US-1676, and US-1680 the smallest (30.0–32.0 cm). The scion/rootstock trunk circumference ratio was not affected by injection treatment but varied significantly among rootstocks. US-1680 produced the highest ratio (0.99), followed by sour orange, US-1673, US-1676, and US-2111 (0.89), while Swingle produced the lowest (0.64). No significant interactions were observed between rootstock cultivar and injection treatment for any measured variables in either year. There were no significant block effects.

### 2.3. Tree Health

Tree health was assessed by visually rating foliar HLB symptoms and canopy density (Table S2). In November 2023, OTC-injected trees showed significantly fewer foliar HLB symptoms (2.8) than control trees (3.1). The rootstock cultivar did not significantly influence symptom ratings. Canopy density ratings did not significantly differ between injection treatments. However, the rootstock cultivar significantly influenced canopy density. US-1688 induced the densest canopy (4.3), while US-2132 induced the least dense canopy (2.2).

**Figure 1:**
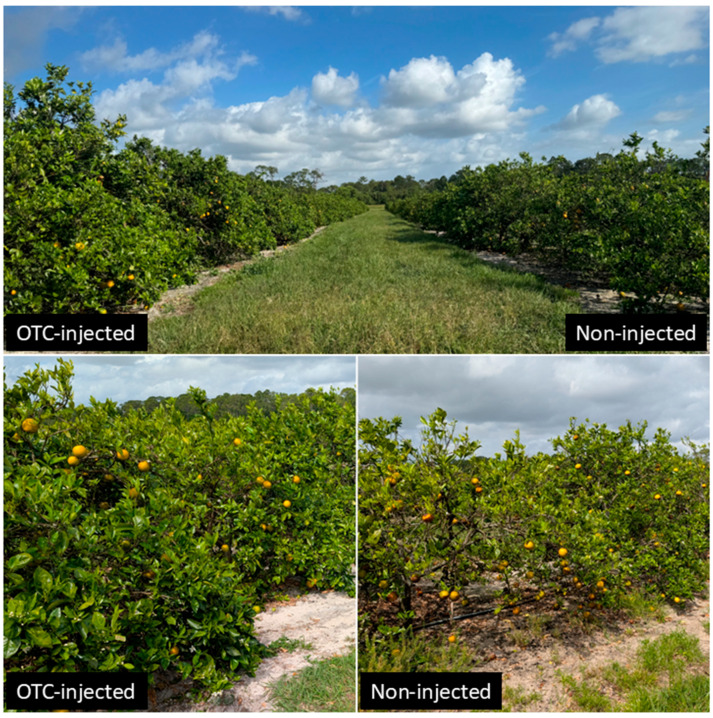
Visual comparison of OTC-injected and non-injected (control) ‘Valencia’ sweet orange trees at the 2025 harvest, following two consecutive years of OTC trunk injection.

In November 2024, the impact of injection treatment on tree health was more pronounced, with significant differences in both foliar HLB symptom ratings and canopy density ratings (Table S2; Figure 1). OTC-injected trees had significantly fewer foliar HLB symptoms (2.6) than control trees (3.4). The rootstock cultivar also significantly influenced symptom ratings. US-1688 induced the least foliar HLB symptoms (2.5), while US-2111, US-2132, and US-2137 induced the most (3.3–3.6). Canopy density was significantly higher in OTC-injected trees (4.0) than in control trees (3.1). Rootstock cultivar also had a significant effect on canopy density. Sour orange and US-1688 induced the densest canopies (4.3 and 4.2, respectively), while US-2132 induced the least dense canopy (2.5). In 2024, a significant interaction between rootstock cultivar and injection treatment was observed for foliar HLB symptom ratings, with control trees displaying a higher symptom severity than OTC-injected trees across most rootstocks. Among the control trees, US-2111 exhibited the most severe foliar symptoms, followed by US-2132 and US-2137. In contrast, US-1688 induced the least foliar HLB symptoms (data not shown). No significant interactions were observed for canopy density ratings in either year. Block effects were not significant in either year.

### 2.4. Pre-Harvest Fruit Drop (%), Fruit Yield, and Yield Efficiency

**Figure 2:**
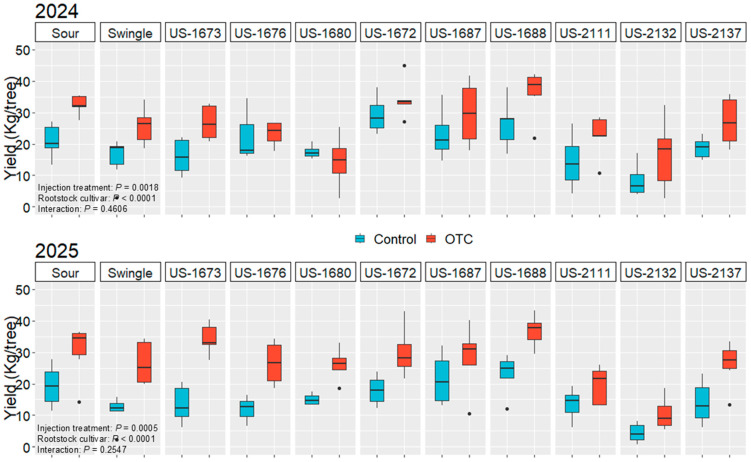
Illustration of yield per tree (kg) of ‘Valencia’ trees on different rootstocks under control and OTC-injected (OTC) treatments at the 2024 (**top**) and 2025 (**bottom**) harvest. Data are presented as boxplots with median, interquartile range, and outliers.

In 2024, pre-harvest fruit drop (%) was significantly affected by injection treatment but not by rootstock cultivar (Table S3). OTC-injected trees had a significantly lower fruit drop rate (3.6%) than control trees (6.2%). Yield was significantly influenced by injection treatment and rootstock cultivar (Table 2, Figure 2). OTC-injected trees had significantly higher yields (26.3 kg/tree) than control trees (19.4 kg/tree), representing a 36% increase. Yield was also significantly influenced by the rootstock. US-1672 and US-1688 induced the highest yields (31.9 kg/tree and 31.4 kg/tree, respectively), while US-2132 induced the lowest (12.5 kg/tree). Yield efficiency exhibited a marginally significant difference (*p* = 0.0562) between injection treatments, with a trend towards higher efficiency in OTC-injected trees (9.0 kg/m^3^) compared to control trees (7.4 kg/m^3^) (Table S3). The rootstock cultivar significantly influenced yield efficiency, with US-2137 exhibiting the highest yield efficiency (13.3 kg/m^3^), significantly greater than the other rootstocks (5.2–8.1 kg/m^3^) except US-1676, US-1672, and US-1688 (9.1–10.3 kg/m^3^). No significant interactions between rootstock cultivar and injection treatment were observed for any of the variables. Block effects were not significant.

In 2025, pre-harvest fruit drop (%) was not significantly affected by injection treatment or rootstock cultivar (Table S3). Yield was significantly influenced by injection treatment and rootstock cultivar (Table 2, Figure 2). OTC-injected trees had significantly higher yields (26.9 kg/tree) than control trees (15.8 kg/tree), representing a 70% increase. Yield varied significantly among rootstocks. US-1688 induced the highest yields (30.1 kg/tree), while US-2132 induced the lowest (7.6 kg/tree) (Figure 2). Yield efficiency was significantly affected by the injection treatment (Table S3). OTC-injected trees had significantly higher yield efficiency (9.5 kg/tree) than control trees (6.3 kg/tree). Yield efficiency varied significantly among rootstocks. US-1673 and US-2137 induced the highest yield efficiency (9.2 kg/m^3^), and US-2132 the lowest (4.7 kg/m^3^). No significant interactions between rootstock cultivar and injection treatment were observed for any of the variables. Block effects were not significant.

### 2.5. Fruit and Juice Quality

In 2024, individual fruit weight was significantly affected by injection treatment and rootstock cultivar (Table 2). OTC-injected trees produced significantly heavier fruits (160 g) than control trees (145 g). Fruit weight also varied significantly among rootstocks. US-2137 produced the heaviest fruit (172 g), while Swingle produced the lightest (131 g). Juice percentage was not significantly influenced by injection treatment but was significantly affected by rootstock cultivar (Table S4). Swingle, US-2132, and US-2137 produced fruit with higher juice percentages (59.9–60.1%) than sour orange, US-1680, US-1672, US-1687, and US-1688 (57.9–58.3%). Juice color was significantly influenced by injection treatment, with OTC-injected trees producing better colored juice (37.1) compared to control trees (36.7) (Table S4). There was no significant difference among rootstocks for the juice color. TSS were significantly higher in OTC-injected trees (9.9 °Brix) than in control trees (8.9 °Brix), representing an 11% increase (Table 2). Rootstock cultivar also significantly affected TSS, with US-2132 and US-2137 producing the most (10.2 and 9.9 °Brix, respectively) and sour orange, US-1680, US-1672, US-1687, and US-1688 the least (9.0–9.1 °Brix). TA was not significantly influenced by the injection treatment but showed significant differences among rootstocks (Table S4). US-2132 induced the highest acidity (0.95%), followed by Swingle and US-2111 (0.87%), while US-1680 produced the lowest (0.72%). Both injection treatment and rootstock cultivar significantly influenced the TSS/TA ratio (Table S4). OTC-injected trees produced a higher ratio (12.5) than control trees (10.9). US-1680 and US-2137 produced higher ratios (12.7 and 12.4, respectively) than US-1688 (11.2) and US-2132 (10.9). The pounds solids per box were also significantly affected by injection treatment and rootstock cultivar (Table S4). OTC-injected trees produced significantly more pounds of solids per box (5.24 lbs) than control trees (4.70 lbs). Swingle, US-2132, and US-2137 produced the most pounds of solids per box (5.31–5.51 lbs), while sour orange, US-1680, US-1673, US-1672, US-1687, and US-1688 produced the least (4.69–4.88 lbs). Significant interactions between rootstock cultivar and injection treatment were observed for TA and TSS/TA, where non-injected US-2132 induced the highest TA (0.98%), significantly greater than the other rootstocks (0.72–0.92%), and injected US-2137 induced the highest TSS/TA (13.7), significantly greater than the other rootstocks (9.6–13.1) (data not shown). Block effects were not significant for any of the measured variables.

In 2025, fruit weight was significantly affected by injection treatment but not by rootstock cultivar (Table 2). OTC-injected trees produced significantly heavier fruits (153 g) than control trees (126 g). Juice percentage was not significantly influenced by injection treatment or rootstock cultivar (Table S5). Injection treatment significantly influenced juice color, with OTC-injected trees producing better colored juice (36.3) than control trees (35.8) (Table S5). The rootstock cultivar did not significantly affect juice color. TSS were significantly higher in OTC-injected trees (9.6 °Brix) than in control trees (8.8 °Brix), representing a 9% increase (Table 2). The rootstock cultivar did not significantly affect TSS. TA was significantly affected by both the injection treatment and the rootstock cultivar (Table S5). Control trees produced a significantly higher TA (0.87%) than OTC-injected trees (0.83%). US-2111 induced the highest TA (0.94%), while sour orange and US-1680 induced the lowest (0.82% and 0.81%, respectively). The TSS/TA ratio was significantly affected by injection treatment, with OTC-injected trees having a higher ratio (11.6) than control trees (10.3) (Table S5). The rootstock cultivar did not significantly influence the TSS/TA ratio. Pounds solids per box were significantly higher in OTC-injected trees (5.21 lbs) than in control trees (4.76 lbs) (Table S5). The rootstock cultivar did not significantly influence pounds of solids per box. No significant interactions between rootstock cultivar and injection treatment were observed for any fruit/juice quality parameter. Block effects were not significant for any of the measured variables.

### 2.6. Fibrous Root Characterization

**Figure 3:**
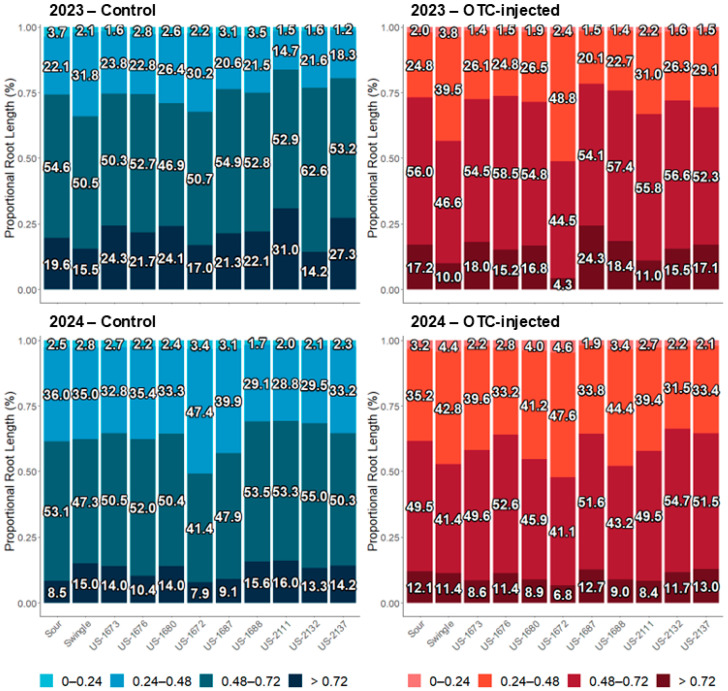
Illustration of the fibrous root distribution of rootstock cultivars across different diameter classes in 2023 (**top**) and 2024 (**bottom**). For statistical details, see Table S6.

In 2023, significant differences were observed between injection treatments and among rootstock cultivars for the proportional root length in the 0.24–0.48 mm, 0.48–0.72 mm, and >0.72 mm diameter classes (Table S6; Figure 3). Injected trees had a significantly higher proportion of fibrous roots in the 0.24–0.48 mm diameter class (29.1%) compared to control trees (23.1%). Among the rootstocks, US-1672 had the highest proportion of roots in this diameter class (39.5%), while US-1687 had the lowest (20.4%). For the 0.48–0.72 mm diameter class, US-2132 had the highest proportion (59.6%), whereas US-1672 and Swingle had the lowest (47.6% and 48.5%, respectively). In the >0.72 mm diameter class, injected trees had a significantly lower proportion of roots (15.3%) than control trees (21.7%), but there was no significant rootstock effect. No significant interaction was observed for any diameter class in 2023.

In 2024, significant differences were observed between injection treatments and among rootstock cultivars for the proportional root length in the 0–0.24 mm, 0.24–0.48 mm, and 0.48–0.72 mm diameter classes (Table S6; Figure 3). Injected trees had a significantly higher proportion of roots in the 0–0.24 mm (3.0%) and 0.24–0.48 mm (38.4%) diameter classes compared to control trees (2.5% and 34.6%, respectively). In contrast, injected trees had a significantly lower proportion of roots in the 0.48–0.72 mm diameter class (48.2%) than control trees (50.4%). Among rootstock cultivars, US-1672 had the highest proportion of fibrous roots in the 0–0.24 mm diameter class (4.0%), while US-2132 and US-2137 had the lowest (2.2% and 2.2%, respectively). US-1672 also had the highest proportion of fibrous roots in the 0.24–0.48 mm diameter class (47.5%), whereas US-2111, US-2132, and US-2137 had the lowest (30.5–34.1%). US-2132 had the highest proportion of roots in the 0.48–0.72 mm diameter class (54.8%), while US-1672 had the lowest (41.2%). No significant interaction was detected for any diameter class in 2024. Block effects were also not significant (data not shown).

### 2.7. Multivariate Analysis

**Figure 4:**
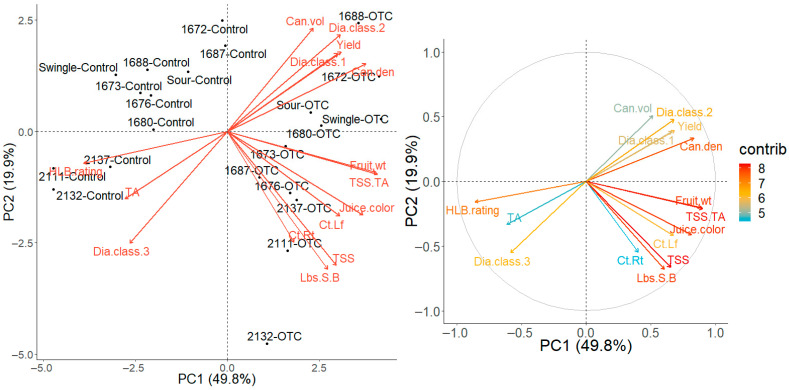
Principal Component Analysis (PCA) biplots representing the relationships between injection treatment, rootstock cultivars, and tree responses in the 2024–2025 production season. The first two principal components (PC1 and PC2) are presented. (**Left Panel**): PCA biplot. (**Right Panel**): contribution plot. The direction and length of the variable loadings (vectors) indicate the degree of correlation between variables and principal components. The contribution plot highlights each variable’s influence on the principal components, with color gradients indicating relative importance. The gray (correlation) circle marks the theoretical maximum length a variable vector can have; vectors that approach the edge of this circle are better represented in the first two principal components.

A PCA was conducted for selected tree response variables that were significantly affected by injection treatment and/or rootstock cultivar in the 2024–2025 production season. These included fruit weight (Fruit.wt), yield (Yield), canopy volume (Can.vol), TA (TA), TSS (TSS), TSS/TA (TSS.TA), juice color (Juice.color), canopy density (Can.den), HLB symptoms foliar ratings (HLB.rating), leaf Ct-value (Ct.Lf), root Ct-value (Ct.Rt), pounds solids per box (Lbs.S.B), and fibrous roots diameter classes 0–0.24 mm, 0.24–0.48, and 0.48–0.72 mm (Dia.class1, Dia.class.2, and Dia.class.3, respectively) (Figure 4). The first two principal components explained 69.7% of the total variance, with 49.8% attributed to PC1 and 19.9% to PC2. Along PC1, trees injected with OTC were separated from non-injected trees, primarily driven by HLB foliar symptoms and fruit/juice quality traits. PC2 captured variability among rootstocks, mainly associated with root Ct-values, juice quality, canopy volume, and fibrous root diameter classes. In the contribution analysis, TSS, TSS/TA, fruit weight, juice color, and pounds solids per box emerged as the variables with the highest contributions to PCAs.

**Figure 5:**
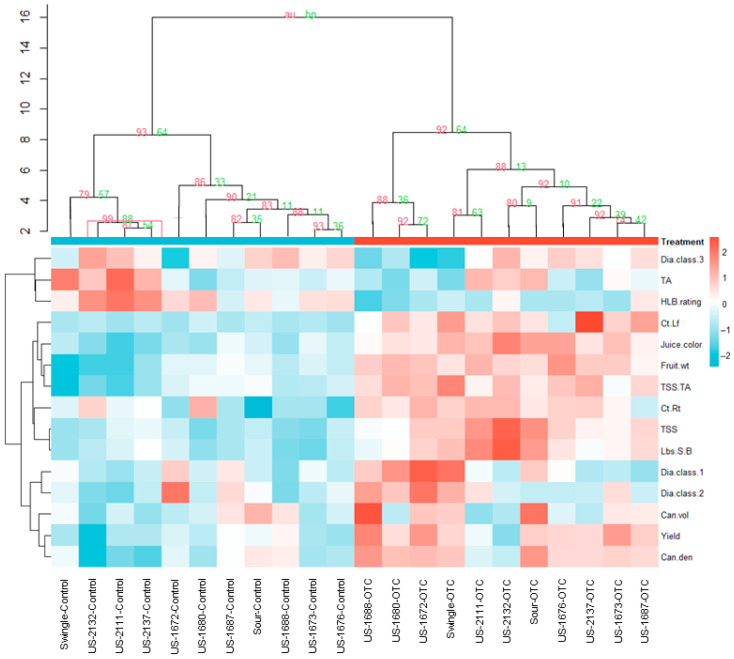
Hierarchical cluster analysis heatmap illustrating relationships of injection treatment and rootstock cultivar based on standardized tree responses measured in the 2024–2025 production season. Rows represent measured response variables, while columns correspond to each rootstock under control (non-injected) or OTC-injected (OTC) conditions. Colors indicate the relative intensity of each trait (z-scores), with red representing higher values and blue representing lower values. Dendrograms show clustering among treatments and response variables, revealing distinct profiles associated with OTC injection and rootstock cultivar. Numbers on branches denote AU (Approximate Unbiased, red) and BP (Bootstrap Probability, green) values (%). The height axis shows the clustering distance.

A hierarchical clustering heatmap analysis also revealed a clear separation between OTC-injected and non-injected trees, while clustering among rootstocks was variable within injection treatments (Figure 5). Within the OTC-injected trees, those grafted on US-1688, US-1680, US-1672, and Swingle formed a cluster separate from the other rootstocks, mainly based on similarities in favorable traits related to fibrous root characteristics, canopy density, and yield. In contrast, within non-injected trees, US-2111, US-2132, US-2137, and Swingle clustered from the other rootstocks mainly based on less favorable traits related to fruit- and juice quality, HLB severity, and larger diameter fibrous roots. These patterns were confirmed by hierarchical clustering of standardized trait means followed by multiscale bootstrap resampling (10,000 replicates), which generated two highly supported groups corresponding to OTC-injected and non-injected (control) trees (AU = 93% and 92%; BP = 64%), indicating that injection treatment was the dominant source of the multivariate structure (Figure 5). Within both the control and OTC-injected group, several subclusters with moderate to high support were observed (AU = 74–99%).

## 3. Discussion

The results from this study demonstrate the effectiveness of an integrated approach to managing HLB by combining rootstock selection with the systemic delivery of oxytetracycline (OTC) via trunk injection. The results underscore the potential of this strategy to sustain citrus productivity and fruit quality in areas severely affected by HLB, like Florida.

A consistent and significant reduction in leaf bacterial titers (higher Ct-values) was observed four months after OTC injection in both years of the study, reinforcing the efficacy of systemically delivered antibiotics for use in plant disease management. This finding is consistent with previous research that demonstrated suppression of *C*Las populations after trunk injection of OTC and other antibiotics [33,34]. Specifically, Archer et al. [26] reported that in mature ‘Valencia’ and ‘Hamlin’ sweet orange trees, OTC trunk injections resulted in a significant increase in Ct-values (reduction in *C*Las titers) in leaves three, six-, and nine-months after injection, with the highest reduction in bacterial titers observed after six months.

While the reduction in bacterial titers in fibrous roots was less pronounced and not statistically significant in the first year, a significant reduction was observed in the second year, suggesting a cumulative effect of repeated OTC applications. Archer et al. [25] reported reductions in root *C*Las titers between three weeks and three months after one OTC injection. Aside from the different time points of assessments, variations among experiments may be due to the uneven distribution of *C*Las within the tree, as previously documented [35], along with the limited translocation of OTC to the root system. In addition to OTC injections, fibrous root *C*Las titers were consistently affected by the rootstock cultivar. Among the evaluated rootstocks, sour orange had the highest bacterial titer in both years, aligning with previous studies that identified sour orange as more susceptible to *C*Las colonization than other rootstocks. For example, Tardivo et al. [36] reported higher fibrous root bacterial titers in field-grown sour orange than other rootstocks such as US-942, US-802, and Swingle under HLB-endemic conditions. Similarly, a greenhouse study found higher fibrous root bacterial titers in sour orange compared to various trifoliate orange hybrid rootstock cultivars [37].

OTC trunk injections significantly improved canopy volume, canopy density, and reduced foliar HLB symptoms (Figure 1), consistent with reports by Archer et al. [25,26]. These improvements are likely attributable to the efficacy of OTC in reducing bacterial load and restoring vascular functioning. Moreover, yield increased across both years in response to the injections. In 2024, OTC-injected trees exhibited a 36% increase in yield compared to controls, and this yield increase was amplified in 2025, resulting in a 70% increase. Yield increases corresponded to reductions in pre-harvest fruit drop, especially in 2024, indicating that OTC improves fruit retention. Previous studies reported similar effects of OTC trunk injections on reducing fruit drop of HLB-affected citrus trees [25,26,27]. However, no differences were found in the pre-harvest fruit drop in the second year. Yield efficiency, defined as fruit yield per unit of canopy volume, was less responsive to the OTC treatment alone but showed significant variation among rootstock cultivars. US-2137 and US-1673 consistently induced higher yield efficiency than more vigorous rootstocks such as sour orange or US-1688, though higher total yields were induced by the latter. The ability to induce compact growth while supporting a favorable fruit-to-canopy ratio may be advantageous for producers favoring high-density plantings [38] and for citrus under protective screen (CUPS) production systems [39].

Except for Swingle, which is known for its low scion/trunk diameter ratio, all rootstock hybrids exhibited ratios of 0.7–0.9. The scion/rootstock circumference ratio represents the smoothness of the graft union and has long been considered an indicator of scion-rootstock compatibility [40]. Ratios approaching 1.0 are most desirable and indicate a higher compatibility, while a smaller or larger ratio reflects an overgrowth of one of the grafting partners. However, this does not necessarily limit trunk health or tree physiology [16]. The ratios for the novel rootstocks identified in this study were similar to those of sour orange, indicating good compatibility with the scion.

Across both years, OTC injections improved most measured fruit and juice quality responses, resulting in larger fruit with better colored juice, a higher soluble solids content, and lower acidity. The positive effects of OTC injections on fruit and juice quality were previously reported [25,26,27]. Rootstock selection also played a key role in shaping juice quality responses. In 2024, US-2132 and US-2137, both hybrids of *C. maxima* × US-942 and Swingle, induced the most TSS. This may be associated with their lower vigor, as small tree-size-inducing rootstocks generally tend to allocate more photoassimilates to reproductive growth rather than vegetative development [41].

Multivariate analyses revealed a strong influence of injection treatment. In the PCA, OTC-injected and non-injected trees separated along PC1 (49.8%) primarily based on tree health and fruit/juice quality traits, while PC2 (19.9%) reflected differences among rootstock cultivars. A hierarchical clustering heatmap showed a similar separation, confirmed by bootstrap clustering, which produced two highly supported groups corresponding to OTC injection and control treatments.

Among the best performing rootstocks in this study were US-1688, US-1687, US-1672, and sour orange, as indicated by the larger canopy volume and yield imparted to the scion, suggesting that vigor may be a critical determinant of grafted tree performance under HLB pressure. These findings align with field observations by Bowman et al. [22,23] and Tardivo et al. [42], where US-1688, US-1672, and other ‘SuperSour’ type rootstocks exhibited vigorous canopy growth traits and higher productivity under HLB-endemic conditions, but in the absence of OTC trunk injections. Notably, US-1688, US-1687, and US-1672 share the same genetic background derived from crosses between *Citrus maxima* and *C. reticulata*, which may contribute to their overall resilience and productivity. Similarly, Rodrigues et al. [13] and Kunwar et al. [20] highlighted the importance of rootstock-induced canopy vigor in mitigating HLB severity and supporting fruit production.

While OTC injections provide substantial benefits to tree health and productivity, uncertainties remain regarding their long-term sustainability, particularly in regard to antimicrobial resistance. Currently, only three antibiotics (streptomycin, oxytetracycline, and kasugamycin) are registered for use in plant agriculture [43]. Despite its widespread use as foliar sprays in other fruit tree systems [44], streptomycin is not currently approved for trunk injection in citrus. Compared with streptomycin, tetracyclines, including OTC, have shown a lower frequency of resistance development [43,44]. Moreover, Glusberger et al. [45] demonstrated a low frequency of spontaneous antimicrobial resistance to OTC in *Liberibacter crescens*, a culturable relative of *C*Las. According to the authors, this, combined with the restrictive vector and pathogen habitats, suggests a low likelihood of fast field resistance development. However, with the increasing adoption of OTC injections to reduce *C*Las bacteria in infected trees, the potential for resistance development cannot be ignored. Alternate therapies have been suggested for use in combination or alternating with OTC, including other natural or synthetic antibacterials, defense inducers, or other molecules [46,47], and are under investigation.

Aside from moderate effects on root bacterial titers, OTC influenced the fibrous root size distribution in both years after injection. This study is the first to report a positive effect of OTC injection on fibrous root size distribution. Specifically, OTC-treated trees exhibited a higher proportion of thinner fibrous roots, possibly indicating improved root health or regeneration associated with the antibiotic. Root size distribution also varied based on the rootstock genetic background. US-1672 had the highest proportion of fine roots in the 0.24–0.48 mm class, while US-2132 had the highest proportion of thicker roots (>0.72 mm). These trends were maintained in the second year, with injected trees again producing more of the finer roots than non-injected trees, and similar genotype-dependent responses. US-1672 continued to be distinct from rootstocks derived from *C. maxima* × US-942 parentage in having a relatively higher proportion of finer roots. Given that HLB compromises nutrient acquisition and root turnover [17,48], rootstock genotypes that maintain fine-root production, such as US-1672, could provide a significant advantage. While enhanced fertilization strategies were shown to have a limited impact on disease progression [49,50], they remain vital in supporting root function and tree productivity [51].

Multivariate structure within each injection treatment revealed shifts in rootstock responses. For example, in both the heatmap and pvclust dendrogram, trees on Swingle clustered with trees on the three *C. maxima* × US-942 hybrids (US-2111, US-2132, US-2137) in the control treatment, driven by less favorable fruit- and juice quality traits and HLB severity. A shift occurred upon OTC injection, resulting in a greater similarity for Swingle between trees on US-1688 and US-1672, driven by fine root traits, canopy density, and yield. This injection treatment-induced shift in performance may be indicative of the true potential of a rootstock in the absence of HLB.

Taken together, the long-term sustainability of citrus production under endemic HLB necessitates the integration of different management practices. Aside from other important practices like vector management and good nutrition, these include the systemic delivery of OTC to reduce pathogen titers and restore tree health and productivity, and the selection of rootstocks with the ability to restrict *C*Las movement, maintain or regenerate fibrous roots, and impart superior horticultural traits, including vigor, to the grafted tree. Future breeding efforts should prioritize these characteristics and optimize scion-rootstock interactions to sustain productivity in infected orchards. Marker-assisted selection (MAS) and genomic selection (GS) offer promising tools for accelerating the identification of these desirable traits from diverse germplasm sources.

## 4. Materials and Methods

### 4.1. Plant Material

Nine new rootstocks developed by the USDA-ARS citrus rootstock breeding program through sexual hybridization were used. In addition to the new hybrids, two commercially available and well-established rootstocks with historical and commercial importance, sour orange (*Citrus aurantium*) and Swingle citrumelo (*C. paradisi* × *Poncirus trifoliata*), were included as standards in this trial. Both sour orange and Swingle citrumelo have been classified as HLB susceptible [9]. The same is true for the parent species of the nine US hybrids, *C. maxima* and *C. reticulata*, while *P. trifoliata* as well as US-942 are considered tolerant [9,19]. Rootstocks and parentages are shown in Table 3. All rootstocks were propagated by stem cuttings as previously described [52] and grafted during spring 2014 using certified budwood of the ‘Valencia’ sweet orange (*C. sinensis*) clone 1-14-19, the most widely used clone in Florida (FDACS, 2023). A total of 115 trees were used for the study. Trees were planted into the field at the USDA research farm (Ft. Pierce, St Lucie County, FL, USA) in October 2014 (latitude 27.437062°, longitude −80.427313°) as single tree replications into double rows on raised beds at a spacing of 2.1 m within and 7.6 m between rows.

The experiment was arranged in a split-plot design, where injection treatment was the main factor and rootstock cultivar the subfactor. The main factor had two levels: (1) OTC-injected and (2) non-injected (control), while the subfactor included 11 rootstock cultivars (Table 3). Some trees died during the early tree establishment phase, resulting in fewer trees for some of the rootstocks. Trees were maintained using standard production practices [23].

### 4.2. Oxytetracycline Trunk Injection

**Figure 6:**
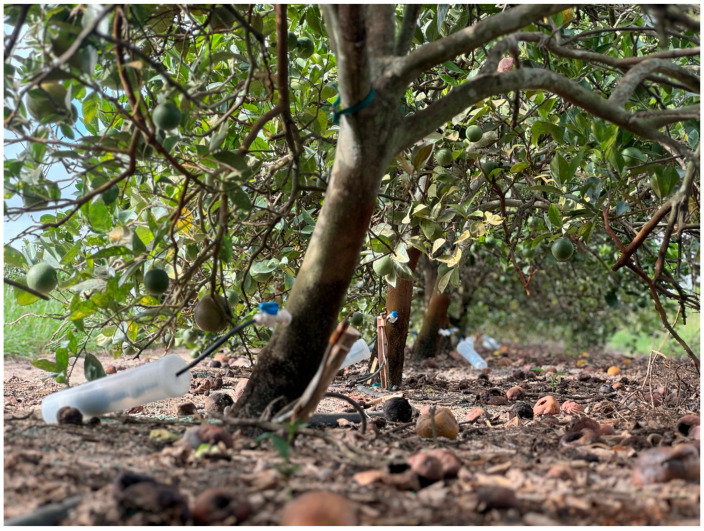
Trunk injection using FlexInject™ injectors for the systemic delivery of OTC.

Half of the trees were injected with an injectable formulation of oxytetracycline hydrochloride (OTC) in July 2023 and 2024, and the other half was left non-injected (control). As each block constituted one row of trees, every other row was injected while the other rows remained non-injected, resulting in 5–6 replicates per rootstock/treatment combination. The OTC formulation used was ReMedium TI^®^ (TJ BioTech LLC, Buffalo, SD, USA; 95.0% oxytetracycline hydrochloride) dissolved in reverse osmosis water acidified with muriatic acid to a pH of 1.8–2.0. Each tree received 0.75 g OTC (a.i.) dissolved in a volume of 75 mL (10,000 ppm). The dose was selected based on the trunk diameter size according to label recommendations and positive results from previous research that used a similar dose per unit trunk diameter [25,26]. Injections were made using FlexInject™ injectors (TJ BioTech LLC, Buffalo, SD, USA) (Figure 6). A 6.75 mm (17/64) brad-point drill bit was used to drill one hole into the scion trunk approximately 5 cm above the graft union and 2.5 cm deep. For year 2, injections were performed on the opposite side to year 1 injections. The injector tips were inserted directly into the hole and were removed once the trees had taken up all the solution. Injections were made on a sunny day between 9 am and 12 pm.

### 4.3. Candidatus Liberibacter asiaticus (CLas) Detection

Leaves and fibrous roots were collected in November of each year (four months after injection). Six leaves per tree were randomly collected from different areas in the canopy. Leaves were collected from the most recent mature flush and stored at −20 °C until analysis. Fibrous roots (≤1.5 mm in diameter) were collected from different areas of the soil under the canopy drip line, washed with water, blotted dry, and stored at −20 °C until analysis. Two leaf punches (4 mm in diameter) were collected from each leaf from the midvein and combined for a total of 12 punches per replicate. Punches were pulverized by freezing with liquid nitrogen and shaking for 3 min with a BioSpec Mini-Beadbeater-96 (Bartlesville, OK, USA). Fibrous roots were pulverized with liquid nitrogen using a mortar and pestle. One hundred milligrams of ground tissue was used for DNA extraction using the DNeasy Plant Pro Kit (Qiagen, Valencia, CA, USA). Real-time PCR assays were performed using primers HLBas/HLBr and probe HLBp, and normalization with primers COXf/COXr and probe COXp, as described in [53]. Amplifications were performed over 40 cycles using the Applied Biosystems QuantStudio 3 Real-Time PCR system (Applied Biosystems, Foster City, CA, USA) and the iTaq Universal Probes Supermix (Bio-Rad, Hercules, CA, USA) according to the manufacturer’s instructions. For samples for which no signal was detected after 40 amplification cycles, a Ct-value of 41 was used for statistical analysis purposes.

### 4.4. Biometric Assessments

#### 4.4.1. Tree Growth

Tree height, canopy volume, and scion and rootstock trunk diameters at 5 cm above and below the graft union were measured in November 2024. In addition, visual ratings of tree health (canopy density and foliar HLB disease symptoms) were conducted in November 2023 and 2024. Canopy density was rated on a scale of 1 to 5, where 1 = very sparse and 5 = very dense. HLB severity was rated on a scale of 1 to 5 where 1 = 0% of branches with HLB symptoms, 2 = 0–25% of branches with HLB symptoms, 3 = 25–50% of branches with HLB symptoms, 4 = 50–75% of branches with HLB symptoms, and 5 = >75% of branches with HLB symptoms. HLB disease symptoms were defined as irregular blotchy mottling of the leaves typical of HLB [4].

#### 4.4.2. Pre-Harvest Fruit Drop, Fruit Yield, and Yield Efficiency

Pre-harvest fruit drop was evaluated one week prior to harvest by counting the number of dropped fruits beneath each tree within each replicated plot. Fruit drop was calculated as the percentage of dropped fruit relative to the number of fruits retained on the tree. Fruit yield was measured at the end of February 2024 and 2025 by counting the number of fruits on each tree at harvest. The yield was estimated based on the average weight per fruit determined from a subset of 40–60 randomly selected fruits per tree (see Section 4.4.3) and expressed as kg of fruit per tree. Yield efficiency was calculated as the ratio of estimated fruit yield to canopy volume (m^3^).

#### 4.4.3. Fruit and Juice Quality

At harvest, a sample of approximately 40–60 randomly collected fruits per tree/replication was collected to assess fruit weight and juice quality responses, including percent juice, juice color, total soluble solids (TSS), and percent titratable acidity (TA). Measurements were conducted at the Juice Processing Pilot Plant, Citrus Research and Education Center, University of Florida, Institute of Food and Agricultural Sciences, Lake Alfred, FL. Fruit weight was measured using a digital scale, and juice was extracted using a pinpoint extractor (JBT, Chalfont, PA, USA). The juice percentage was calculated based on the extracted juice. Juice color was assessed using a spectrophotometer (Macbeth Color-EYE 3100). TSS was measured in degrees Brix (°Brix) following standard commercial procedures (John Bean Technologies Corporation, 2018). TA was determined by titrating juice samples with NaOH, using phenolphthalein as an indicator, and the TSS/TA was determined. The soluble solids per box of fruit were calculated (https://www.flrules.org/gateway/ruleno.asp?id=20-61.0071 (accessed on 18 September 2025), with one box corresponding to 90 pounds (40.8 kg) of fruit.

### 4.5. Fibrous Root Characterization

Fibrous roots (<1.5 mm in diameter) were collected in November 2023 and 2024 from different areas of the soil under the canopy, washed with water, and blotted dry. Roots were scanned using a flatbed scanner (Epson Perfection V850 Pro; Epson America, Los Alamitos, CA, USA) at 400 dpi resolution. Scanned images were processed using RhizoVision Explorer v1.0 [54] to determine the proportional length of roots in four diameter classes: 0–0.24 mm, 0.24–0.48 mm, 0.48–0.72 mm, and >0.72 mm.

### 4.6. Statistical Analysis

All analyses were conducted using R version 4.3 [55]. Prior to conducting the analysis of variance (ANOVA), data were tested for normality (Shapiro–Wilk test for residuals) and homogeneity of variance to ensure compliance with model assumptions. When model assumptions were not met, variables were transformed (log) or analyzed with a non-parametric approach, as specified below. A linear mixed model was used with injection treatment and rootstock cultivar as fixed factors and block as a random factor. Models were fit with the linear mixed model lme4/lmerTest. When significant differences were detected (*p* < 0.05), pairwise comparisons of estimated marginal means, adjusted to Tukey’s method (Tukey’s Honest Significant Difference, HSD), were performed using emmeans. Visual ratings of tree health were analyzed using an aligned ranks transformation (ARTool) ANOVA to account for non-parametric data distributions.

*Principal Component Analysis (PCA).* Principal component analysis (PCA) was performed on standardized variables (z-scores) to mitigate the influence of different measurement scales among variables and to explore the multivariate structure [56]. Contribution analysis was included to assess the relative importance of individual variables. PCA was performed using the FactoMineR package [57]. Variables included in the PCA were selected based on their statistical significance.

*Heatmap hierarchical clustering and bootstrapping.* The trait means for each rootstock × injection treatment combination were standardized (z-scores) and displayed in a heatmap using Euclidean distance and Ward.D2 linkage for both rows and columns, to visualize similarity patterns across injection treatments and rootstocks. To assess the stability of clusters, we applied multiscale bootstrap resampling with 10,000 replicates using pvclust [58]. For each node in the dendrogram, we report AU (approximately unbiased) and BP (bootstrap probability) values. Values closer to 100% indicate a higher stability of a cluster.

## 5. Conclusions

This research provides strong evidence for the efficacy of an integrated approach to managing HLB, combining the genetic tolerance of rootstocks with the antimicrobial action of systemically delivered OTC. Under HLB pressure, this combined strategy improved tree health, productivity, and fruit quality. Among the tested rootstocks, US-1688 and US-1672 emerged as the best performers, mainly due to their vigor and higher productivity compared to the other rootstocks. These results underscore the role of rootstock selection and support the combined adoption of genetic and therapeutic strategies to enhance the resilience and sustainability of citrus production systems.

## Figures and Tables

**Table 1:** *Candidatus* Liberibacter asiaticus (*C*Las) detection of OTC-injected and non-injected (control) ‘Valencia’ trees on different rootstocks.

Factor	2023		2024	
	Leaf Ct-Values	Fibrous Roots Ct-Values	Leaf Ct-Values	Fibrous Roots Ct-Values
*Injection treatment*				
OTC-injected	28.1 ± 0.77 a	33.3 ± 0.66	28.5 ± 0.69 a	31.9 ± 0.54 a
Control	22.2 ± 0.79 b	31.8 ± 0.66	21.6 ± 0.73 b	29.1 ± 0.57 b
*p*-value	<0.0001	0.0722	<0.0001	0.0002
*Rootstock cultivar*				
Sour orange	25.4 ± 1.59	29.3 ± 1.21 b	23.9 ± 1.60	27.2 ± 1.24 b
Swingle	23.7 ± 1.43	34.2 ± 1.16 ab	26.6 ± 1.65	30.9 ± 1.28 a
US-1673	26.1 ± 1.52	31.1 ± 1.26 ab	25.1 ± 1.59	29.6 ± 1.23 ab
US-1676	24.7 ± 1.67	33.4 ± 1.41 ab	21.6 ± 1.94	29.2 ± 1.49 ab
US-1680	25.0 ± 1.63	33.8 ± 1.31 ab	26.0 ± 1.76	32.6 ± 1.38 ab
US-1672	24.0 ± 1.52	30.2 ± 1.16 ab	23.9 ± 1.86	30.7 ± 1.31 ab
US-1687	22.5 ± 1.58	32.3 ± 1.27 ab	26.3 ± 1.78	28.7 ± 1.38 ab
US-1688	26.4 ± 1.36	31.1 ± 1.10 ab	23.3 ± 1.51	30.3 ± 1.17 ab
US-2111	25.1 ± 1.36	33.8 ± 1.10 ab	24.2 ± 1.60	31.5 ± 1.25 ab
US-2132	25.5 ± 1.43	34.6 ± 1.11 a	25.3 ± 1.52	33.3 ± 1.17 a
US-2137	28.0 ± 1.30	34.2 ± 1.05 ab	29.5 ± 1.53	31.6 ± 1.13 ab
*p*-value	0.3426	0.0064	0.0873	0.0308
*Rootstock cultivar* × *Injection treatment*				
*p*-value	0.2284	0.2778	0.1915	0.4876
*Block*				
*p*-value	0.8996	0.5279	1.0000	0.5915

*C*Las detection was performed four months after injection. Values represent mean ± standard error. Different letters within columns indicate significant differences according to Tukey’s honestly significant difference test at *p* ≤ 0.05. Letters are not shown when *p* ≤ 0.05.

**Table 2:** Yield, fruit weight, and TSS of OTC-injected and non-injected (control) ‘Valencia’ trees on different rootstocks.

Factor	2024			2025		
	Yield (kg/Tree)	Fruit Weight (g)	TSS	Yield (kg/Tree)	Fruit Weight (g)	TSS
*Injection treatment*						
OTC-injected	26.3 ± 1.39 a	160 ± 3.6 a	9.9 ± 0.08 a	26.9 ± 1.45 a	153 ± 3.8 a	9.6 ± 0.12 a
Control	19.4 ± 1.42 b	145 ± 3.7 b	8.9 ± 0.08 b	15.8 ± 1.42 b	126 ± 3.8 b	8.8 ± 0.12 b
*p*-value	0.0018	0.0024	<0.0001	0.0005	<0.0001	0.0002
*Rootstock cultivar*						
Sour orange	26.8 ± 2.12 ab	154 ± 4.6 ab	9.0 ± 0.13 c	24.9 ± 1.98 ab	145 ± 5.9	9.1 ± 0.18
Swingle	20.6 ± 2.17 bcd	131 ± 5.0 d	9.8 ± 0.14 ab	19.2 ± 2.12 b	123 ± 6.7	9.2 ± 0.21
US-1673	21.4 ± 2.27 abcd	159 ± 5.2 ab	9.2 ± 0.14 bc	24.2 ± 2.21 ab	143 ± 7.1	8.9 ± 0.22
US-1676	23.0 ± 2.75 abcd	168 ± 6.2 ab	9.1 ± 0.18 bc	19.8 ± 2.67 ab	148 ± 8.7	9.3 ± 0.27
US-1680	16.0 ± 2.56 cd	158 ± 5.8 ab	9.1 ± 0.16 c	20.7 ± 2.48 ab	145 ± 7.5	9.0 ± 0.23
US-1672	31.9 ± 2.39 a	162 ± 5.4 ab	9.0 ± 0.15 c	26.0 ± 2.33 ab	145 ± 7.46	9.3 ± 0.23
US-1687	26.5 ± 2.50 abc	147 ± 5.7 bcd	9.0 ± 0.16 c	25.4 ± 2.43 ab	142 ± 7.8	9.2 ± 0.24
US-1688	31.4 ± 2.16 a	146 ± 4.9 bcd	9.0 ± 0.14 c	30.1 ± 2.11 a	140 ± 6.7	8.9 ± 0.21
US-2111	18.3 ± 2.16 bcd	151 ± 5.0 bc	9.8 ± 0.14 ab	16.7 ± 2.20 bc	134 ± 7.0	9.6 ± 0.22
US-2132	12.5 ± 2.17 d	132 ± 5.0 cd	10.2 ± 0.14 a	7.6 ± 2.26 c	130 ± 7.2	9.6 ± 0.22
US-2137	22.9 ± 2.07 abc	172 ± 4.8 a	9.9 ± 0.13 a	20.1 ± 2.02 b	138 ± 6.4	9.3 ± 0.20
*p*-value	<0.0001	<0.0001	<0.0001	<0.0001	0.2924	0.2282
*Rootstock cultivar* × *Injection treatment*						
*p*-value	0.4606	0.6463	0.0623	0.2547	0.3802	0.6577
*Block*						
*p*-value	0.5450	0.6883	1.0000	0.4727	0.4996	1.0000

Values represent mean ± standard error. Different letters within columns indicate significant differences according to Tukey’s honestly significant difference (HSD) test at *p* ≤ 0.05. Letters are not shown when *p* > 0.05.

**Table 3:** Rootstocks cultivar parentages.

Rootstock	Parentage
Sour orange	*C. aurantium*
Swingle	*C. paradisi* ‘Duncan’ × *P. trifoliata*
US-1673	*C. maxima* ‘Hirado’ × *C. tachibana*
US-1676	*C. maxima* ‘Hirado’ × *C. tachibana*
US-1680	*C. maxima* ‘Hirado’ × *C. tachibana*
US-1672	*C. maxima* ‘Hirado’ × *C. reticulata* ‘Cleopatra’
US-1687	*C. maxima* ‘Hirado’ × *C. reticulata* ‘Cleopatra’
US-1688	*C. maxima* ‘Hirado’ × *C. reticulata* ‘Cleopatra’
US-2111	*C. maxima* ‘Hirado’ × US-942 (*C. reticulata* ‘Sunki’ × *Poncirus trifoliata* ‘Flying Dragon’)
US-2132	*C. maxima* ‘Hirado’ × US-942
US-2137	*C. maxima* ‘Hirado’ × US-942

## Data Availability

The original contributions presented in this study are included in the article/Supplementary Materials. Further inquiries can be directed to the corresponding authors.

## Supplementary Materials

The following supporting information can be downloaded at: https://www.mdpi.com/article/10.3390/10.3390/plants14193020/s1, Table S1: Tree height, canopy volume, scion and rootstock trunk circumference, and scion/rootstock trunk circumference ratio of OTC-injected and non-injected (control) ‘Valencia’ trees on different rootstocks in November 2024. Table S2: Tree health ratings of OTC-injected and non-injected (control) ‘Valencia’ trees on different rootstocks. Table S3: Fruit drop (%) and yield efficiency of OTC-injected and non-injected (control) ‘Valencia’ trees on different rootstocks. Table S4: Fruit and juice quality of OTC-injected and non-injected (control) ‘Valencia’ trees on different rootstocks in 2024. Table S5: Fruit and juice quality of OTC-injected and non-injected (control) ‘Valencia’ trees on different rootstocks in 2025. Table S6: Proportional root length of different diameter classes fibrous roots of OTC-injected and non-injected (control) ‘Valencia’ trees on different rootstocks across different diameter classes.
