# Supplementary material for: Field Performance of Novel Citrus Rootstocks Grafted with ‘Valencia’ Orange and Their Response to Systemic Delivery of Oxytetracycline
Source: Plants (Basel). 2025 Sep 29;14(19):3020. doi: 10.3390/plants14193020 (PMC12526186; doi:10.3390/plants14193020)
Supplement: Supplementary file 1 [file plants-14-03020-s001.zip › plants-3808617-supplementary.pdf]

Supplemental Tables

Table S1. Tree height, canopy volume, scion and rootstock trunk circumference, and scion/rootstock trunk circumference ratio of OTC-injected and non-injected (control) 'Valencia' trees on different rootstocks in November 2024.

Factor	Height (m)	Canopy volume (m^3)	Scion trunk circ. (cm)	Rootstock trunk circ. (cm)	Scion/Rootstock circ. ratio
Injection treatment					
OTC-injected	2.0 ± 0.04	3.1 ± 0.14 a	30.0 ± 0.44	35.8 ± 0.59	0.85 ± 0.01
Control	2.0 ± 0.04	2.7 ± 0.13 b	28.7 ± 0.46	34.4 ± 0.63	0.84 ± 0.01
P-value	0.1568	0.0328	0.0585	0.1052	0.4033
Rootstock cultivar					
Sour orange	2.3 ± 0.09 a	3.8 ± 0.30 a	34.0 ± 1.01 a	38.2 ± 1.29 bc	0.89 ± 0.02 b
Swingle	2.1 ± 0.09 ab	3.1 ± 0.31 ab	28.2 ± 1.04 c	44.6 ± 1.32 a	0.64 ± 0.02 e
US-1673	1.9 ± 0.09 ab	2.7 ± 0.29 ab	28.5 ± 1.00 c	32.0 ± 1.27 d	0.89 ± 0.02 ab
US-1676	2.0 ± 0.11 ab	2.7 ± 0.36 ab	26.4 ± 1.21 c	30.1 ± 1.54 d	0.89 ± 0.03 ab
US-1680	1.9 ± 0.10 ab	2.5 ± 0.33 ab	29.5 ± 1.12 abc	30.0 ± 1.42 d	0.99 ± 0.02 a
US-1672	1.9 ± 0.09 ab	3.0 ± 0.31 ab	30.0 ± 1.07 abc	34.1 ± 1.35 bcd	0.88 ± 0.02 b
US-1687	2.1 ± 0.09 ab	3.1 ± 0.31 ab	30.4 ± 1.12 abc	35.5 ± 1.41 bcd	0.86 ± 0.02 bc
US-1688	2.2 ± 0.08 a	3.9 ± 0.28 a	33.4 ± 0.95 ab	38.8 ± 1.21 ab	0.86 ± 0.02 bc
US-2111	1.9 ± 0.09 ab	2.4 ± 0.30 b	29.1 ± 1.01 bc	32.7 ± 1.29 cd	0.89 ± 0.02 b
US-2132	1.9 ± 0.08 b	2.2 ± 0.28 b	27.1 ± 0.95 c	35.9 ± 1.21 bcd	0.76 ± 0.02 d
US-2137	1.9 ± 0.08 b	2.3 ± 0.27 b	26.2 ± 0.92 c	34.2 ± 1.17 bcd	0.78 ± 0.02 cd
P-value	0.0004	<0.0001	<0.0001	<0.0001	<0.0001
Rootstock cultivar × Injection treatment					
P-value	0.6361	0.3638	0.4770	0.5484	0.5669
Block					
P-value	1.0000	1.0000	1.0000	0.9759	1.0000

Values represent mean ± standard error. Different letters within columns indicate significant differences according to Tukey's honestly significant difference (HSD) test at $P \leq 0.05$. Letters are not shown when $P > 0.05$.

Table S2. Tree health ratings of OTC-injected and non-injected (control) 'Valencia' trees on different rootstocks.

Factor	2023		2024	
	**Foliar HLB symptoms**	**Canopy density**	**Foliar HLB symptoms**	**Canopy density**
Injection treatment				
OTC-injected	2.8 ± 0.09 b	3.6 ± 0.15	2.6 ± 0.09 b	4.0 ± 0.10 a
Control	3.1 ± 0.09 a	3.5 ± 0.15	3.4 ± 0.09 a	3.1 ± 0.10 b
P-value	0.0018	0.2223	<0.0001	<0.0001
Rootstock cultivar				
Sour orange	3.1 ± 0.16	3.5 ± 0.21 bc	2.9 ± 0.20 ab	4.3 ± 0.21 a
Swingle	2.9 ± 0.15	3.7 ± 0.21 abc	3.0 ± 0.20 ab	3.8 ± 0.22 ab
US-1673	3.0 ± 0.15	3.7 ± 0.20 abc	2.9 ± 0.19 ab	3.5 ± 0.21 abc
US-1676	3.0 ± 0.18	3.6 ± 0.24 abc	2.9 ± 0.24 ab	3.5 ± 0.26 abc
US-1680	3.1 ± 0.17	3.2 ± 0.22 cd	3.0 ± 0.22 ab	3.5 ± 0.24 abc
US-1672	2.8 ± 0.16	3.7 ± 0.21 abc	2.9 ± 0.21 ab	3.9 ± 0.22 ab
US-1687	2.9 ± 0.18	4.3 ± 0.23 ab	3.0 ± 0.22 ab	3.7 ± 0.22 ab
US-1688	2.8 ± 0.14	4.3 ± 0.19 a	2.5 ± 0.18 b	4.2 ± 0.20 a
US-2111	3.1 ± 0.14	3.2 ± 0.19 cd	3.4 ± 0.20 a	2.9 ± 0.21 bc
US-2132	3.4 ± 0.15	2.2 ± 0.20 d	3.6 ± 0.19 a	2.5 ± 0.20 c
US-2137	2.8 ± 0.14	3.3 ± 0.18 bc	3.3 ± 0.18 a	3.2 ± 0.19 bc
P-value	0.2342	<0.0001	0.0034	<0.0001
Rootstock cultivar × *Injection treatment*				
P-value	0.8969	0.5815	0.0261	0.6227

Values represent mean ± standard error. Variables were analyzed using the Aligned Rank Test. Different letters within columns indicate significant differences according to Tukey's honestly significant difference test. Letters are not shown when $P > 0.05$. Ratings were conducted on a scale of 1 to 5, with 1 = best and 5 = worst for foliar HLB symptoms and 1 = worst and 5 = best for canopy density.

Table S3. Fruit drop (%) and yield efficiency of OTC-injected and non-injected (control) 'Valencia' trees on different rootstocks.

Factor	2024		2025	
	**Fruit drop (%)**	**Yield efficiency (kg/m^3)**	**Fruit drop (%)**	**Yield efficiency (kg/m^3)**
Injection treatment				
OTC-injected	3.6 ± 0.63 b	9.0 ± 0.65	8.7 ± 1.29	9.5 ± 0.96 a
Control	6.2 ± 0.65 a	7.4 ± 0.63	11.3 ± 1.27	6.3 ± 0.95 b
P-value	0.0193	0.0562	0.1235	0.0406
Rootstock cultivar				
Sour orange	5.5 ± 1.29	6.4 ± 1.52 b	9.8 ± 1.88	6.0 ± 1.35 ab
Swingle	5.1 ± 0.95	7.1 ± 1.10 b	8.5 ± 2.10	6.8 ± 1.13 ab
US-1673	5.7 ± 0.95	8.1 ± 1.10 b	7.8 ± 2.10	9.2 ± 1.13 a
US-1676	5.2 ± 1.15	10.3 ± 1.35 ab	7.6 ± 2.55	8.2 ± 1.33 ab
US-1680	2.8 ± 1.07	6.4 ± 1.25 b	10.2 ± 2.37	8.9 ± 1.25 ab
US-1672	3.5 ± 1.0	9.7 ± 1.17 ab	12.1 ± 2.22	9.3 ± 1.18 ab
US-1687	4.6 ± 1.10	6.5 ± 1.29 b	8.1 ± 2.31	9.5 ± 1.21 ab
US-1688	4.1 ± 0.91	9.1 ± 1.05 ab	7.9 ± 2.00	8.3 ± 1.09 ab
US-2111	5.3 ± 0.91	7.9 ± 1.05 b	11.4 ± 2.00	7.8 ± 1.13 ab
US-2132	8.1 ± 0.91	5.2 ± 1.06 b	16.4 ± 2.01	4.7 ± 1.15 b
US-2137	3.8 ± 0.87	13.3 ± 1.00 a	10.5 ± 1.92	9.2 ± 1.05 a
P-value	0.065	<0.0001	0.0675	0.0041
Rootstock cultivar × Injection treatment				
P-value	0.2308	0.3030	0.4276	0.5182
Block				
P-value	1.0000	0.7528	0.2128	0.9679

Values represent mean ± standard error. Different letters within columns indicate significant differences according to Tukey's honestly significant difference (HSD) test at $P \leq 0.05$. Letters are not shown when $P > 0.05$.

Table S4. Fruit and juice quality of OTC-injected and non-injected (control) 'Valencia' trees on different rootstocks in 2024.

Factor	Juice (%)	Juice color	TA (% citric acid)	TSS/TA	Pounds solids per box
Injection treatment					
OTC-injected	58.7 ± 0.22	37.1 ± 0.06 a	0.80 ± 0.01	12.5 ± 0.17 a	5.24 ± 0.04 a
Control	59.0 ± 0.21	36.7 ± 0.06 b	0.82 ± 0.01	10.9 ± 0.17 b	4.70 ± 0.04 b
P-value	0.2894	0.0010	0.1364	<0.0001	<0.0001
Rootstock cultivar					
Sour orange	57.9 ± 0.28 b	36.7 ± 0.09	0.75 ± 0.02 cd	12.0 ± 0.26 ab	4.69 ± 0.07 c
Swingle	60.0 ± 032 a	37.2 ± 0.10	0.87 ± 0.02 b	11.5 ± 0.29 ab	5.31 ± 0.07 a
US-1673	58.8 ± 0.32 ab	36.9 ± 0.11	0.82 ± 0.02 bc	11.4 ± 0.30 ab	4.88 ± 0.08 c
US-1676	58.9 ± 0.38 ab	37.0 ± 0.13	0.78 ± 0.02 bcd	11.8 ± 0.37 ab	4.85 ± 0.10 bc
US-1680	57.9 ± 0.38 b	37.0 ± 0.12	0.72 ± 0.02 d	12.7 ± 0.34 a	4.73 ± 0.09 c
US-1672	58.2 ± 0.32 b	36.8 ± 0.11	0.76 ± 0.02 cd	11.9 ± 0.32 ab	4.70 ± 0.08 c
US-1687	58.1 ± 0.35 b	36.9 ± 0.12	0.79 ± 0.02 bcd	11.5 ± 0.34 ab	4.69 ± 0.09 c
US-1688	58.3 ± 0.30 b	36.8 ± 0.10	0.81 ± 0.02 bcd	11.2 ± 0.29 b	4.75 ± 0.07 c
US-2111	59.1 ± 0.30 ab	36.9 ± 0.10	0.87 ± 0.02 b	11.4 ± 0.29 ab	5.23 ± 0.07 ab
US-2132	59.9 ± 0.32 a	36.7 ± 0.10	0.95 ± 0.02 a	10.9 ± 0.29 b	5.51 ± 0.07 a
US-2137	60.1 ± 0.29 a	36.9 ± 0.10	0.80 bcd	12.4 ± 0.28 a	5.35 ± 0.07 a
P-value	<0.0001	0.0517	<0.0001	0.0007	<0.0001
Rootstock cultivar × Injection treatment					
P-value	0.1920	0.0948	0.0405	0.0077	0.2157
Block					
P-value	1.0000	1.0000	0.3046	0.3917	1.0000

Values represent mean ± standard error. Different letters within columns indicate significant differences according to Tukey's honestly significant difference (HSD) test at $P \leq 0.05$. Letters are not shown when $P > 0.05$.

Table S5. Fruit and juice quality of OTC-injected and non-injected (control) 'Valencia' trees on different rootstocks in 2025.

Factor	Juice (%)	Juice color	TA (% citric acid)	TSS/TA	Pounds solids per box
Injection treatment					
OTC-injected	60.1 ± 0.23	36.3 ± 0.07 a	0.83 ± 0.01 b	11.6 ± 0.17 a	5.21 ± 0.07 a
Control	60.0 ± 0.22	35.8 ± 0.07 b	0.87 ± 0.01 a	10.3 ± 0.17 b	4.76 ± 0.07 b
P-value	0.8325	0.0002	0.0267	0.0002	0.0003
Rootstock cultivar					
Sour orange	60.5 ± 0.34	36.1 ± 0.09	0.82 ± 0.02 b	11.2 ± 0.26	4.93 ± 0.11
Swingle	59.9 ± 0.39	36.2 ± 0.11	0.87 ± 0.02 ab	10.7 ± 0.30	4.96 ± 0.12
US-1673	59.6 ± 0.40	36.2 ± 0.11	0.85 ± 0.03 ab	10.6 ± 032	4.81 ± 0.13
US-1676	59.6 ± 0.49	36.2 ± 0.13	0.84 ± 0.03 ab	11.1 ± 0.39	4.98 ± 0.16
US-1680	60.4 ± 0.43	36.0 ± 0.12	0.81 ± 0.03 b	11.2 ± 0.34	4.88 ± 0.14
US-1672	60.2 ± 0.43	36.0 ± 0.12	0.84 ± 0.03 ab	11.2 ± 0.34	5.05 ± 0.13
US-1687	60.0 ± 0.44	36.1 ± 0.12	0.84 ± 0.03 ab	11.1 ± 0.35	4.96 ± 0.14
US-1688	59.5 ± 0.38	35.9 ± 0.11	0.83 ± 0.02 ab	10.8 ± 0.30	4.77 ± 0.12
US-2111	60.5 ± 0.40	35.9± 0.11	0.94 ± 0.03 a	10.3 ± 0.32	5.22 ± 0.13
US-2132	60.8 ± 0.41	36.2 ± 0.11	0.90 ± 0.03 ab	10.7 ± 0.32	5.28 ± 0.13
US-2137	60.0 ± 0.37	35.9 ± 0.10	0.86 ± 0.02 ab	11.0 ± 0.29	5.02 ± 0.12
P-value	0.3102	0.3735	0.0330	0.5591	0.1100
Rootstock cultivar × Injection treatment					
P-value	0.0522	0.2344	0.2300	0.1626	0.3031
Block					
P-value	1.0000	1.0000	1.0000	0.5985	1.0000

Values represent mean ± standard error. Different letters within columns indicate significant differences according to Tukey's honestly significant difference (HSD) test at $P \leq 0.05$. Letters are not shown when $P > 0.05$.

Table S6. Proportional root length of different diameter classes fibrous roots of OTC-injected and non-injected (control) 'Valencia' trees on different rootstocks across different diameter classes.

**Factor**	**2023**				**2024**			
	**0–0.24 mm**	**0.24–0.48 mm**	**0.48–0.72 mm**	**> 0.72 mm**	**0–0.24 mm**	**0.24–0.48 mm**	**0.48–0.72 mm**	**> 0.72 mm**
Injection treatment								
OTC-injected	1.9 ± 0.21	29.1 ± 0.31 a	53.7 ± 0.95	15.3 ± 1.56 b	3.0 ± 0.15 a	38.4 ± 0.20 a	48.2 ± 0.76 b	10.4 ± 0.96
Control	2.3 ± 0.22	23.1 ± 0.33 b	52.9 ± 0.97	21.7 ± 1.59 a	2.5 ± 0.15 b	34.6 ± 0.23 b	50.4 ± 0.78 a	12.5 ± 0.98
P-value	*0.2741*	*0.0006*	*0.7902*	*0.0036*	*0.0048*	*0.0109*	*0.0183*	*0.0740*
Rootstock cultivar								
Sour orange	2.8 ± 0.48	23.5 ± 2.98 bc	55.3 ± 2.16 ab	18.4 ± 3.55	2.9 ± 0.34 ab	35.6 ± 2.76 ab	51.3 ± 1.74 ab	10.3 ± 2.20
Swingle	3.0 ± 0.48	35.7 ± 2.98 ab	48.5 ± 2.16 b	12.8 ± 3.55	3.6 ± 0.34 ab	38.9 ± 2.76 ab	44.4 ± 1.74 bc	13.2 ± 2.20
US-1673	1.5 ± 0.48	24.9 ± 2.98 bc	52.4 ± 2.16 ab	21.2 ± 3.55	2.4 ± 0.34 ab	36.2 ± 2.76 ab	50.0 ± 1.74 ab	11.3 ± 2.20
US-1676	2.1 ± 0.59	23.8 ± 3.60 abc	55.6 ± 2.61 ab	18.4 ± 4.29	2.5 ± 0.41 ab	34.3 ± 3.33 ab	52.3 ± 2.10 ab	10.9 ± 2.66
US-1680	2.2 ± 0.54	26.5 ± 3.34 abc	50.8 ± 2.42 ab	20.5 ± 3.97	3.2 ± 0.38 ab	37.2 ± 3.08 ab	48.1 ± 1.95 abc	11.4 ± 2.46
US-1672	2.3 ± 0.51	39.5 ± 3.16 a	47.6 ± 2.29 b	10.7 ± 3.77	4.0 ± 0.36 a	47.5 ± 2.93 a	41.2 ± 1.85 c	7.3 ± 2.34
US-1687	2.3 ± 0.54	20.4 ± 3.34 c	54.5 ± 2.42 ab	22.8 ± 3.97	2.5 ± 0.38 ab	36.8 ± 3.08 ab	49.8 ± 1.95 abc	10.9 ± 2.46
US-1688	2.4 ± 0.48	22.1 ± 2.98 bc	55.1 ± 2.16 ab	20.3 ± 3.55	2.6 ± 0.33 ab	36.8 ± 2.64 ab	48.4 ± 1.67 abc	12.3 ± 2.11
US-2111	1.9 ± 0.48	22.8 ± 2.98 bc	54.3 ± 2.16 ab	21.0 ± 3.55	2.3 ± 0.34 ab	34.1 ± 2.76 b	51.4 ± 1.74 ab	12.2 ± 2.20
US-2132	1.6 ± 0.46	23.9 ± 2.86 bc	59.6 ± 2.07 a	14.9 ± 3.40	2.2 ± 0.33 b	30.5 ± 2.64 b	54.8 ± 1.67 a	12.5 ± 2.11
US-2137	1.4 ± 0.44	23.7 ± 2.72 bc	52.8 ± 1.97 ab	22.2 ± 3.24	2.2 ± 0.31 b	33.3 ± 2.52 b	50.9 ± 1.59 ab	13.6 ± 2.01
P-value	*0.2922*	*0.0002*	*0.0070*	*0.2559*	*0.0045*	*0.0212*	*<0.0001*	*0.8455*
Rootstock cultivar × Injection treatment								
P-value	*0.1938*	*0.3543*	*0.3495*	*0.6484*	*0.0847*	*0.2637*	*0.2850*	*0.7005*

Values represent mean ± standard error. Different letters within columns indicate significant differences according to Tukey's honestly significant difference (HSD) test at $P \leq 0.05$. Letters are not shown when $P > 0.05$.
